# From the design to implementation: the case of the Health Care Integration Councils (CIRA) in Chile

**DOI:** 10.1186/1475-9276-11-S1-A2

**Published:** 2012-01-23

**Authors:** Oscar Arteaga, Alejandra Fuentes, Olga Toro, Alex Alarcón

**Affiliations:** 1 School of Public Health, University of Chile, Santiago de Chile, Chile; 2 Hospital Santiago Oriente Dr. Luis Tisné, Santiago de Chile, Chile

## Background

**Figure 1:**
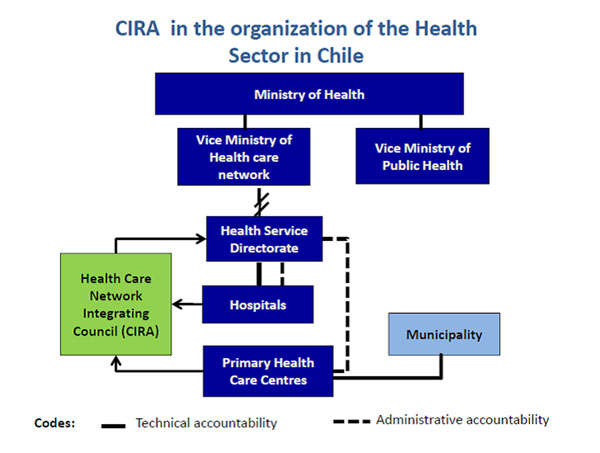


In Chile, primary health care is under municipal administration since 1981 [1]. Hospitals are under administration of geographical health services. This separation has been deemed as an obstacle for an integral functioning of the health care network [2]. In the 2005 health reform in Chile, an administrative arrangement called Health Care Integration Council (CIRA) was set. CIRAs were created in each one of the 29 geographic Health Services (HS), with the purpose of facilitating the development of collaborative relationships among providers within the health care network (see figure 1). The legal framework that regulates CIRA considers the inclusion of private providers and does not include community organisations. The purpose of this study was to explore the views of relevant stakeholders belonging to CIRA on the process of moving from design to implementation of the CIRA policy.

## Material and methods

Thirty five semi-structured interviews were carried out to members of CIRA belonging to a purposive sample of six HS in different regions of the country. All the interviews were recorded after participants’ informed consent. Interviews were performed and analyzed by the group of researchers using Grounded Theory.

## Results

Stakeholders’ perceptions are that CIRAs have been implemented in ways that depart from what was designed in the regulatory framework. This is particularly evident in relation to CIRA members, selection procedures and partially regarding to CIRA role. The regulatory framework is not explicit about the selection of their members. CIRAs were implemented in each HS differently: from non-participatory and rather authoritarian appointment of members to democratic election of some of them. In the regulatory framework CIRA had an advisory role and according to interviewees’ general perceptions, this role is being accomplished. However, the view of some stakeholders is that, in practice, this role has turned CIRA into a structure to exchange information only. Interviewees recognize important contributions coming from CIRA policy. Regardless the ways in which CIRAs were implemented, stakeholders agree to see them as an institutionalized space within the health care network. All levels of health care acknowledge CIRA existence, the issues that are discussed, and respect the decisions taken in this Council.

## Conclusions

Although CIRA has been implemented differently from that stated in norms when it was created (e.g. integrating members), CIRA is valued as a relevant institution within the health care network.
